# Optimized Extraction of cAMP From Jujube by Ultra-High Pressure Technology and the Anti-allergic Effect for Peanut Allergy Mouse

**DOI:** 10.3389/fnut.2022.862900

**Published:** 2022-05-26

**Authors:** Chaowei Sang, Qiao Bai, Xiaoping Feng, Chunyu Wu, Ye Liu, Zhenpeng Gao, Fangyu Long

**Affiliations:** ^1^College of Food Science and Enginering, Northwest A&F University, Xianyang, China; ^2^Beijing Key Lab of Plant Resource Research and Development, Beijing Technology and Business University, Beijing, China

**Keywords:** jujube, cyclic adenosine monophosphate, ultra-high pressure extraction, peanut allergy, Balb/c mouse model

## Abstract

Jujube contains abundant cyclic adenosine monophosphate (cAMP). In contrast, the extraction technology of cAMP from jujube is still to be explored. In this study, the ultra-high pressure extraction (UHPE) conditions for obtaining the maximum cAMP yield from jujube were optimized. Orthogonal array design (OAD) was applied to evaluate the effects of three variables (pressure, pressure-holding time, and liquid-to-solid ratio) by UHPE on cAMP yield. The results showed that the optimal cAMP yield (1223.2 μg/g) was derived at 300 MPa, 20 min duration, and a liquid-to-solid ratio of 2.5 ml/g. In addition, as an important functional ingredient in jujube, cAMP has potential anti-allergic effect. To develop the functional characteristics of jujube, the effect of cAMP was characterized *in vivo* with the Balb/c mouse model of peanut allergy, which was established by subcutaneous injection of crude peanut protein extract (PN). The results showed that treatment with cAMP in PN-sensitized mice suppressed the lesions in jejunal tissues and allergic symptoms and restored spleen index. Meanwhile, cAMP treatment reduced serum levels of specific immunoglobulin E (IgE), histamine, as well as interleukin-4 (IL-4) and stimulated the secretion of tumor necrosis factor-α (TNF-α), whereas the serum levels of interleukin-10 (IL-10) were not affected. Our results suggested that cAMP has an anti-allergic effect in PN-sensitized mice.

## Introduction

Jujube (*Ziziphus jujuba* Mill.), a thorny Rhamnaceous plant, is indigenous to China and widely distributed in the temperate and subtropical areas of the northern hemisphere, especially the arid areas of North China (1). Jujube fruits are rich in various nutrients such as polysaccharides, fiber, vitamin C, cyclic adenosine monophosphate (cAMP), mineral substances of iron and potassium, and phenolics (2). It has also been used as a traditional Chinese medicine for thousands of years with its numerous beneficial effects for human health (3). Moreover, jujube also has various healthcare functions such as antitumor (4), anti-inflammatory (5), hepatoprotective (6), antioxidant (7), and immune-enhancing effects (8).

Cyclic adenosine monophosphate is one of the most important functional ingredients in jujube (9). It was found that the content of cAMP in mature jujube was 30–160 μg/g, the highest of that observed in more than 180 natural plants (10). The cAMP is important for the regulation of intracellular metabolism, fulfilling many biological functions as second messengers. For example, it has positive effect on activating cAMP-dependent protein kinase in myocardial intracellular and substrate proteins (11). It has been proved that exogenous cAMP could inhibit the growth of cancer cells, dilate the blood vessels, improve the liver function, promote the regeneration of nerves, and regulate the metabolism of substances and antiarrhythmic drugs (12).

The traditional solvent extraction method is usually time-consuming and laborious and has low selectivity and/or low extraction yield (13). Many new extraction methodologies have been developed to separate cAMP from jujube. Ultra-high pressure extraction (UHPE) is performed with water as the medium at pressures ranging usually from 100 to 800 MPa (14). This technology can be applied in the food industry relevant to the preservation and processing of food, including the destruction of microorganisms, inactivation of enzyme activity, control of phase changes, altered conformation of biopolymers, and extraction of bioactive ingredients from natural resources (15). The basic working principle of UHPE is that high pressure and rapid pressure relief cause cell deformation and cell wall damage, which result in fast permeation of the solvent into cells and more rapid dissolution of bioactive compounds in the dissolvent (16). Therefore, UHPE usually results in much higher cAMP yields because of the increased mass transfer rate compared to traditional extraction methods. UHPE has been applied in the extraction of flavonoids from jujube leaves (17); however, it has never been used in the extraction of cAMP from jujube.

Cyclic adenosine monophosphate, as an important functional ingredient in jujube, is helpful to better understand the healthcare functions of jujube. It is necessary to study the healthcare effect of cAMP. Recent epidemiologic studies suggest that the estimated North American population prevalence of reported food allergy is 3.9 to 8% in infants and children and 6.6 to 10% in adults (18). Among food allergies, peanut allergy is one of the most common and severe allergies worldwide with an increasing prevalence over the past decades (19). The report shows that peanut allergy accounts for about 30% of all the food allergies (20). Peanut allergy has become a major health concern worldwide, especially in developed countries. The prevalence of peanut allergy among children in the United Kingdom, North America, and Australia has doubled in 10 years and is approximately 1.8, 1.4, and 3.0%, respectively (21). Peanut allergy can produce severe reactions and unlike milk and egg allergy, it is usually lifelong (22). Currently, there are no approved treatments for peanut allergy and the standard of care for peanut allergy is still strict dietary avoidance of peanut and peanut-containing foods (23). The current pharmaceutical intervention is limited to provide immediate relief of symptoms from accidental exposure. Although desensitization of allergic individuals with exposure to subclinical doses of allergens is a common practice for many allergies, it is not suitable for peanut allergy because of the high risk of anaphylactic response to even very low doses of peanut allergen (24). A major focus of current study is the development of disease-modifying treatments that modulate the allergic immune response, protecting against accidental exposure (25). Heijink et al. (26) and some other studies (27, 28) found that cAMP had a beneficial effect on modulating the allergic immune response. At present, however, the literature on the effects of cAMP on allergies is limited.

Currently, relevant study on UHPE for extracting cAMP from jujube and the effect of cAMP on peanut allergy has not been reported. Therefore, it is necessary to optimize cAMP extraction processing parameters for UHPE treatment and systematically study the effect of cAMP on peanut allergy *in vivo*. This study aimed to provide a new cAMP extraction method with a focus on higher extraction yield and efficiency than the traditional methods and develop the functional characteristics of jujube.

## Materials and Methods

### Chemicals and Reagents

The Bicinchoninic Acid (BCA) Protein Quantitative Kit was purchased from Kangwei Century Biotechnology Corporation Ltd. (Beijing, China). ELISA kit was purchased from Win BioTechnique Corporation Ltd. (Shanghai, China). Cyclic AMP (cAMP) standard, 99.9% purity, was purchased from Sigma-Aldrich (USA). Chemical reagents for high-performance liquid chromatography (HPLC) were HPLC grade. Other reagents and chemicals utilized were of an analytical reagent grade.

### Materials

The dried Chinese jujube (*Zizyphus jujuba Mill. cv. Goutouzao*) was purchased from Qingjian in the Shaanxi province of China and then immediately transported to the laboratory, which was uniform in shape and free from disease or any visible blemishes. The jujube with seeds removed was cut into pieces and then crushed into a powder (Q-250B, Shanghai Bingdu Co., Ltd., Shanghai, China) at 2500 r/s for 2 min and sieved through a 100-mesh screen. The obtained sample was stored in a sterile polypropylene pouch at 4°C in a refrigerator before the experiments.

### Animals

Six-week-old Balb/c female mice (18–20 g) were purchased from Xi'an Branch of Chongqing Biotechnology Corporation Ltd. (Chongqing, China). All the animals were raised in our animal facility, which were maintained at a temperature of 20 ± 2°C with a relative humidity of 50–70% under a 12-h light/dark cycle. Mice were maintained under hygienic conditions with free access to food and water, which were fed a special diet without test protein. All the procedures were performed in accordance with the guidelines established by the Northwest A&F University Animal Care and Use Committee.

### Preparation of Crude Peanut Protein Extract

Unshelled raw peanut used in this study was purchased from local market in Yangling, Shaanxi province. Crude peanut protein extract (PN) was prepared as described previously (29). The BCA Protein Quantitative Kit was used to determine the concentration of crude peanut protein.

### Ultra-High Pressure Extraction

The UHPE process was performed using an ultra-high pressure system (SHPP-8.8L, Shanxi Sanshuihe Technology Corporation Ltd., Shanxi, China) with a cylindrical pressure chamber capacity of 8.8 L (30). Distilled water was used as the pressure-transmitting medium. The rate of pressure increase was about 130 MPa/min and the pressure released immediately (<3 s). The treatment time reported in this study did not include the pressure-increase time and pressure-release time. The pressure levels and pressure-holding time were continuously recorded during the pressurization cycle.

Briefly, 20 g of dried jujube powdered sample was accurately weighed and mixed with 100 ml distilled water and then each mixture was vacuum packed (DZ-280/2SD, Guangdong Zhongshan Xianbao Food Corporation Ltd., Guangdong, China) and extracted at different operational conditions (according to Table 1). The extraction process was autocontrolled by computer software. After depressurization, the remaining impurities were removed from the extracted solutions using a centrifuge (HC-3018R, Anhui Zhongke Zhongjia Scientific Instrument Corporation Ltd., Anhui, China) at 5,000 rpm for 20 min. The supernatants were separately collected and then passed through 0.45 μm filters (YY8-1-88, Xi'an Lihe Biological Technology Corporation Ltd., Xi'an, China). Filtrates were stored in a dark ambient glass bottle at 4°C prior to analysis.

**Table 1:** The orthogonal experiment design and results of ultra-high pressure extraction (UHPE).

**No**.	**Factors**			**The yield of cAMP (μg/g)**
	**A**	**B**	**C**	
1	100	20	2.5	1196.84 ± 8.55^a^
2	100	40	5.0	890.57 ± 7.63^d^
3	100	60	10.0	624.81 ± 5.93^h^
4	200	20	5.0	824.65 ± 7.34^e^
5	200	40	10.0	649.75 ± 5.84^g^
6	200	60	2.5	828.22 ± 7.09^e^
7	300	20	10.0	784.03 ± 6.45^f^
8	300	40	2.5	1020.10 ± 8.90^b^
9	300	60	5.0	945.62 ±7.65^c^
K_1_	2712.33	2805.72	3045.39	
K_2_	2302.59	2560.47	2660.64	
K_3_	2749.74	2398.86	2058.66	
k_1_	904.11	935.24	1015.13	
k_2_	767.53	853.49	886.88	
k_3_	916.58	799.62	686.22	
R	149.04	144.44	337.67	

*A, Extraction pressure (MPa).*

*B, Pressure-holding time (min).*

*C, Ratio of liquid to solid (mL/g).*

*K, Sum of yield for the factors at each level.*

*k, The mean values of yield for the factors at each level.*

*R refers to the result of extreme analysis.*

*Yield values are mean ± standard deviation of three determinations.*

*Values with different letters (a–h) in same column represent that they are significantly different from each other*.

### Optimization of Ultra-High Pressure Extraction

The Taguchi experimental design approach has been used for optimization of extraction variables. It is a robust methodology against uncontrollable environmental changes (also known as noise factors), as is the case for raw material variability (31). An orthogonal L_9_(3)^4^ test design in the UHPE group was used to investigate the optimal extraction condition of cAMP from jujube on the basis of the single-factor test. As seen from Table 1, the extraction experiment was carried out with 3 factors and 3 levels, which were set as follows: A: extraction pressure (100, 200, 300 MPa); B: pressure-holding time (20, 40, 60 min); C: ratio of liquid to solid (2.5:1, 5:1, 10:1, v/w).

### High-Performance Liquid Chromatography Analysis

The analysis of the cAMP content in the jujube fruits was determined by high-performance liquid chromatography (HPLC) (CBM-20A, Shimadzu, Japan). The chromatographic column used was XTerra MS C18 (4.6 cm × 250 mm, 5 μm, Waters, Massachusetts, USA), the ratio of mobile phase was V (methanol)/V (0.05 mol/l potassium dihydrogen phosphate) = 1:9, flow rate was 1.0 ml/min, the temperature was 40°C, and the UV detector was UV 259 nm × 0.2 absorbance unit full scale (AUFS). The cAMP standard solutions of different concentrations were prepared and each 20-μL sample was injected into the chromatography column. The integrated area of cAMP chromatographic peaks was recorded and the standard curve was drawn. The extraction yield of cAMP (μg/g) was calculated as the amount of the extracted cAMP (μg) per gram of jujube samples.

### Experimental Protocols and Sample Collection

**Figure 1:**
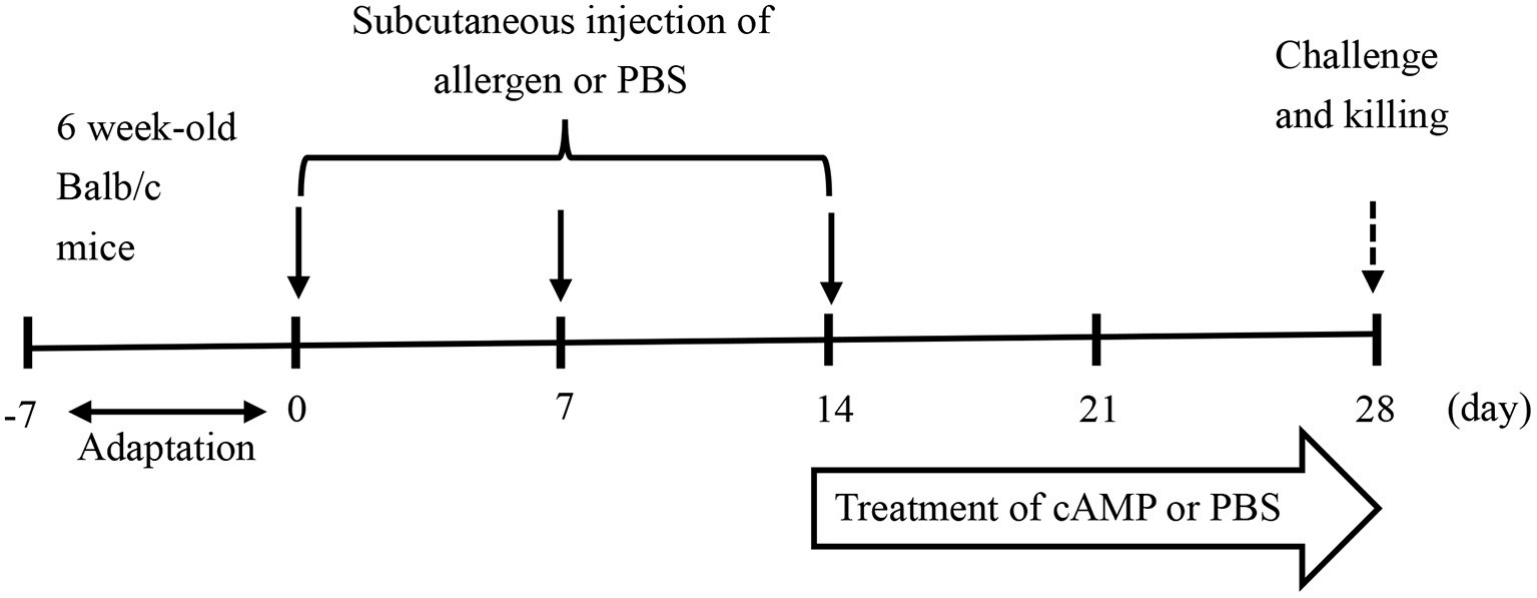
Experimental design of the mouse model of food allergy to peanut. The female Balb/c mice aged 6 weeks were sensitized three times by subcutaneous injections of crude peanut protein extract (PN) (200 μg/mouse) plus adjuvant of Al(OH)_3_ and challenged with PN. In parallel, mice were treated with intragastric cyclic adenosine monophosphate (cAMP) or phosphate-buffered saline (PBS) for 2 weeks.

The female Balb/c mice aged 6 weeks were followed a 1-week acclimatization period and then randomly divided into the three groups, i.e., the control (CK) group, the allergy model (PN) group, and the cAMP-treated (cAMP) group, with 7 mice per group. Mice in the PN and cAMP groups were given subcutaneous injections of 200 μg of PN in a total volume of 200 μl of phosphate-buffered saline (PBS) solution containing 1 mg Al(OH)_3_ per mouse once per week for a duration of 3 weeks. Mice in the CK group received equal amounts of Al(OH)_3_ in PBS solution. 3 weeks later, mice in the cAMP group were treated with intragastric cAMP at the dose of 1.28 mg/kg body weight in 200 μl of PBS solution per mouse three times per week for a duration of 2 weeks. During the same period, the CK group and the PN group received equal amounts of 200 μl of PBS solution in the same way as a control (Figure 1). After the last challenge, fecal samples were collected within an hour and then blood samples were collected from the retro-orbital plexus and serum samples were obtained by centrifugation at 5,000 × g for 20 min at 4°C, which were pooled in equal volumes within each group to determine the serum-specific immunoglobulin E (IgE), histamine, tumor necrosis factor-α (TNF-α), interleukin-4 (IL-4), and interleukin-10 (IL-10) levels. Mice were also sacrificed to collect gut segments from the small intestine, which were snap frozen in liquid nitrogen and then stored at−80°C for later analysis of allergic lesions and treatment effects. At the same time, spleens of mice were collected and weighed.

### Clinical Symptoms

Clinical symptoms of mice were observed during the experiment and anaphylactic scores were determined using a scoring system as described previously (32). The scores were as follows: 0 indicated no signs; 1 indicated repeated scratching; 2 indicated puffiness around the eyes and snout, diarrhea, pilar erecti, and reduced activity with increased respiratory rate; 3 indicated wheezing, labored respiration, and cyanosis; 4 indicated tremor and convulsions; and 5 indicated death.

### Morphological Examination of Jejunum

Jejunal slices were made to evaluate the morphological variation by microscopic inspection as previously described with some modification (33). In brief, the harvested jejunum was cut into 5 mm sections and fixed in 10% buffered formalin for 24 h. After that, proper segments were selected for further dehydrating by preservation in ethanol for 2 h. Then, jejunal segments were cleared in xylene for 20 min, embedded in paraffin wax at 52–58°C, and cut into 5 μm thick sections. Afterward, jejunal segments were stained with H&E for microscopic observation. All the measurements were conducted, in a blinded manner, under light microscopy at 40 × or 400 × magnification.

### Measurement of Spleen Index

The spleen index was measured to assess any immune function alteration in mice during the experiment. The mice were sacrificed and spleens were removed and weighed immediately. The spleen index was calculated according to the following formula, which was described previously (34):


$$\begin{array}{l} \text{Spleen index }\left(\text{mg}/\text{g}\right)=\text{Spleen weight}/\text{Body weight} \end{array}$$

### Measurements of Serum-Specific Immunoglobulin E, Histamine, and Cytokine Levels

The commercial kit of ELISA was used to determine the levels of specific IgE, histamine, TNF-α, IL-4, and IL-10 in serum of mice according to the manufacturer's instructions.

### Statistical Analysis

Results were presented as means ± SDs. The data were analyzed using the SPSS version 25.0 (SPSS Incorporation, Chicago, USA) for ANOVA. The Duncan's test was used for multiple comparisons. Differences with *p*-values of less than 0.05 were considered as statistically significant.

## Results and Discussion

### Effect of Ultra-High Pressure Extraction Condition on Cyclic Adenosine Monophosphate Yield

#### Effect of Extraction Pressure

To determine the optimal extraction conditions of UHPE, three factors, including pressure, pressure-holding time, and the ratio of liquid-to-solid that could affect the effectiveness of UHPE, were considered as major factors (14). The effect of each individual factor was first investigated by single-factor experiments. The experimental procedures and results were presented as follows.

**Figure 2:**
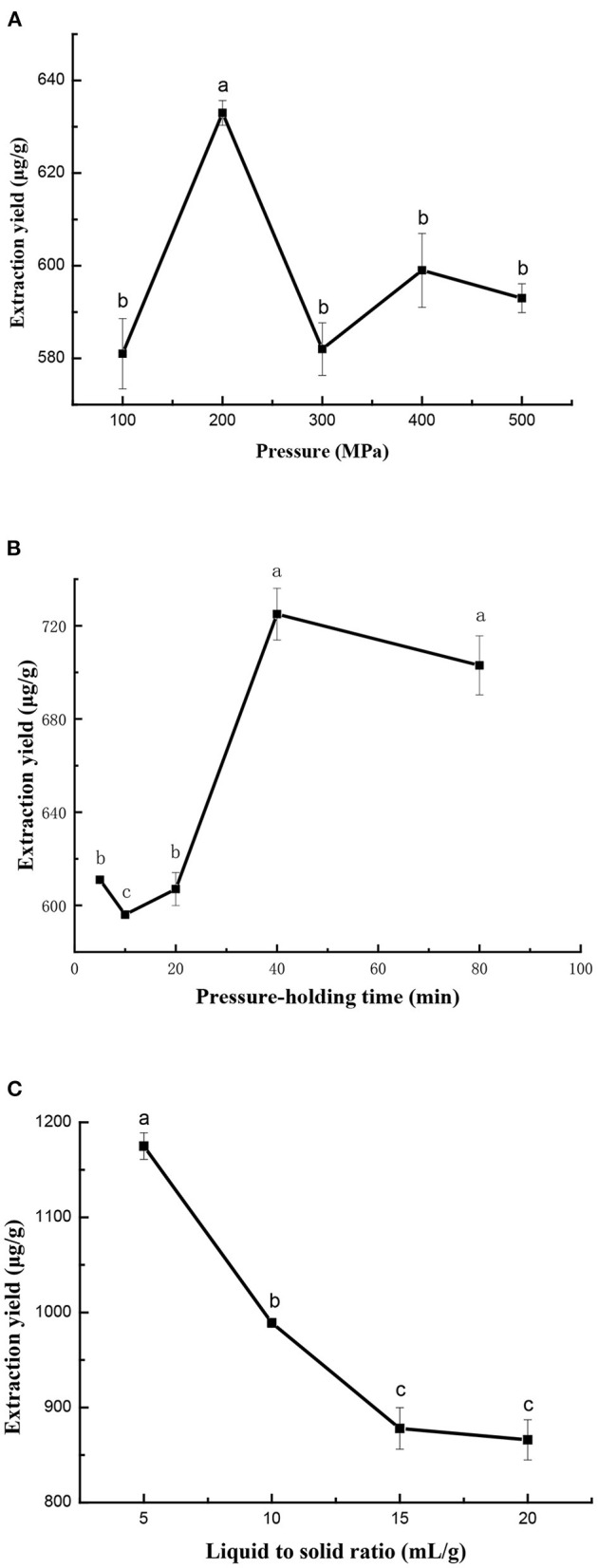
Effect of extraction pressure **(A)**, pressure-holding time **(B)**, and ratio of liquid-to-solid **(C)** on ultra-high pressure extraction (UHPE) yield of cAMP from jujube. **(A)** Pressure-holding time: 10 min; ratio of liquid-to-solid: 15 ml/g. **(B)** Extraction pressure: 200 MPa; ratio of liquid-to-solid: 15 ml/g. **(C)** Extraction pressure: 200 MPa; pressure-holding time: 40 min.

In this study, high pressures from 100 to 500 MPa were applied with a constant ratio of liquid-to-solid of 15 ml/g and pressure-holding time of 10 min. As seen in Figure 2A, the extraction yield of cAMP was greatly influenced by pressure levels. It was initially increased with increasing the pressure from 100 to 200 MPa, whereas subsequently decreased with increasing pressure. Although studies have found that the yields of bioactive compounds increase with the increase in pressure at a range of 600 MPa (35), other studies have obtained similar results to ours. Zhang et al. reported a decrease in flavonoid yield for Xinjiang jujube treated at higher pressure (400 MPa) (17). Thus, an excessively high pressure might damage the target compounds and led to a slight decreasing in the cAMP yield (30). Therefore, the pressure of 200 MPa was considered as the best extraction pressure level.

#### Effect of Pressure-Holding Time

The effect of pressure-holding time on extraction yield of cAMP from jujube is shown in Figure 2B. First, pressure-holding time was set at 5, 10, 20, 40, and 80 min, while other extraction parameters were given as follows: extraction pressure 200 MPa and ratio of liquid-to-solid 15 ml/g. The yields were increased when the extraction time increased from 5 to 40 min and then the yields were almost unchanged from 40 to 90 min. It was well recognized that in UHPE, the pressure transfers to the whole material was a uniform and instant process and the extraction could finished in a shorter time (15). Therefore, a pressure-holding time of 40 min was enough to attain the equilibrium of mass transfer during UHPE.

#### Effect of Ratio of Liquid-to-Solid

The effect of ratio of liquid-to-solid on extraction yield of cAMP from jujube is shown in Figure 2C, while extraction pressure and pressure-holding time were fixed at 200 MPa and 40 min. With an increase in liquid-to-solid ratio from 5 to 20, the yield of cAMP first decreased and then stabilized. It was obvious that the liquid-to-solid ratio was useful for improving the extraction yield of cAMP. But, if the extraction was carried out under a higher liquid-to-solid ratio, the concentration of cAMP in the extraction solution was low. The liquid-to-solid ratio of 5 ml/g was sufficient to reach the high extraction yield and it was used in further experiments.

So, in this study, we adopted extraction pressure of 100–300 MPa, pressure-holding time of 20–60 min, and the liquid-to-solid ratio of 2.5–10 ml/g for further study objects in the orthogonal array design (OAD) experiment.

#### Optimization of Ultra-High Pressure Extraction Conditions and Validation of the Model

To verify whether the effects of individual factors (extraction pressure, pressure-holding time, and ratio of liquid-to-solid) on UHPE efficiency are statistically significant, an ANOVA was used to interpret the experimental data obtained from the OAD optimization. In this study, all the selected factors were examined using an orthogonal L_9_(3)^4^ test design. The total evaluation index was used to analysis by statistical method. The analysis results of OAD and extreme difference (R-value) analysis are given in Table 1. Although the maximum yield of cAMP was 1196.84 μg/g, we could not select the best extraction conditions only based on these outcomes in Table 1 and a further orthogonal analysis was wanted. As seen in Table 1, the impact of the variables on extraction yield of cAMP followed the following order: C (ratio of liquid-to-solid) > A (extraction pressure) > B (pressure-holding time) according to the R-values. The most significant parameter was found to be the ratio of liquid-to-solid, followed by extraction pressure. In contrast, there was no significant difference in pressure-holding time. The order was in good accordance with the order based on the values of F in variance analysis (data not shown). Based on this analysis and considering the cAMP extraction efficiency, the cost of energy, and the feasibility of experiment, the optimum conditions of extraction were, therefore, determined as follows: A_3_B_1_C_1_ (extraction pressure 300 MPa, ratio of liquid-to-solid 2.5 ml/g, and pressure-holding time 20 min). Through confirmatory test, we got the highest yield of cAMP (1223.29 μg/g).

At present, systematic studies on the extraction of cAMP from jujube are limited. Previous studies have shown that the yields of cAMP obtained by water soaking extraction and ultrasonic-assisted extraction are 210 and 268 μg/g, respectively, which are much lower than the yield obtained in this paper (36). Our results were in accordance with many other studies on extraction of bioactive compounds by UHPE, including polyphenols from green tea leaves, pectin from navel orange peel, procyanidins extraction from lychee pericarp, etc. (15, 16, 37). These definitely illustrated that UHPE as a novel method could be utilized in the extraction of many bioactive compounds from various natural materials with less time consumption and higher efficiency.

### Effect of Cyclic Adenosine Monophosphate on Peanut-Sensitized Mice

#### Alleviated Clinical Symptoms

**Figure 3:**
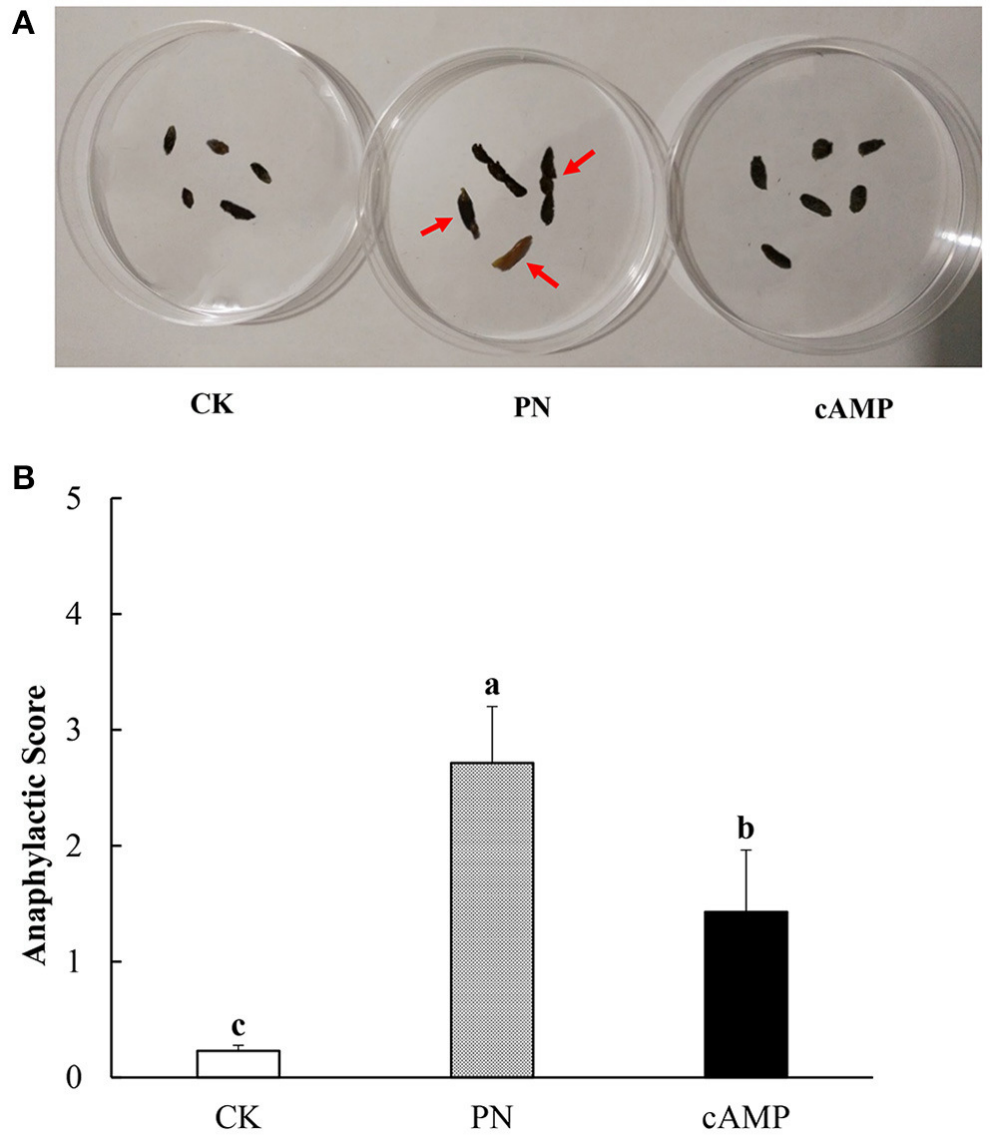
Effect of oral administration with cAMP on clinical symptoms in PN-sensitized mice. **(A)** Morphological images of fecal samples. Fecal samples were collected within an hour of the last challenge. There were the CK group, the PN group, and the cAMP group from left to right. The red arrow indicates typical lesions. **(B)** Anaphylactic score. Data are expressed as the means ± SDs. Different lowercase letters represent mean significant difference (*p* < 0.05) between the groups of mice.

The Balb/c mice are suitable for the study of food allergy for producing highly efficient IgE after being stimulated by allergen (38). In this study, repeated administration of PN led to higher anaphylactic scores than the CK group with symptoms of allergy in the PN group, including scratching and rubbing around the snout and head and diarrhea (Figure 3A). However, as anaphylaxis symptoms progressed after treating PN-sensitized mice with cAMP, anaphylactic scores dropped significantly (Figure 3B). The results showed that repeated oral administration of cAMP could alleviate PN-induced allergic symptoms in mice. It was initially demonstrated that a certain amount of cAMP had an anti-allergic effect on PN-induced allergy.

#### Improved Jejunal Morphology

**Figure 4:**
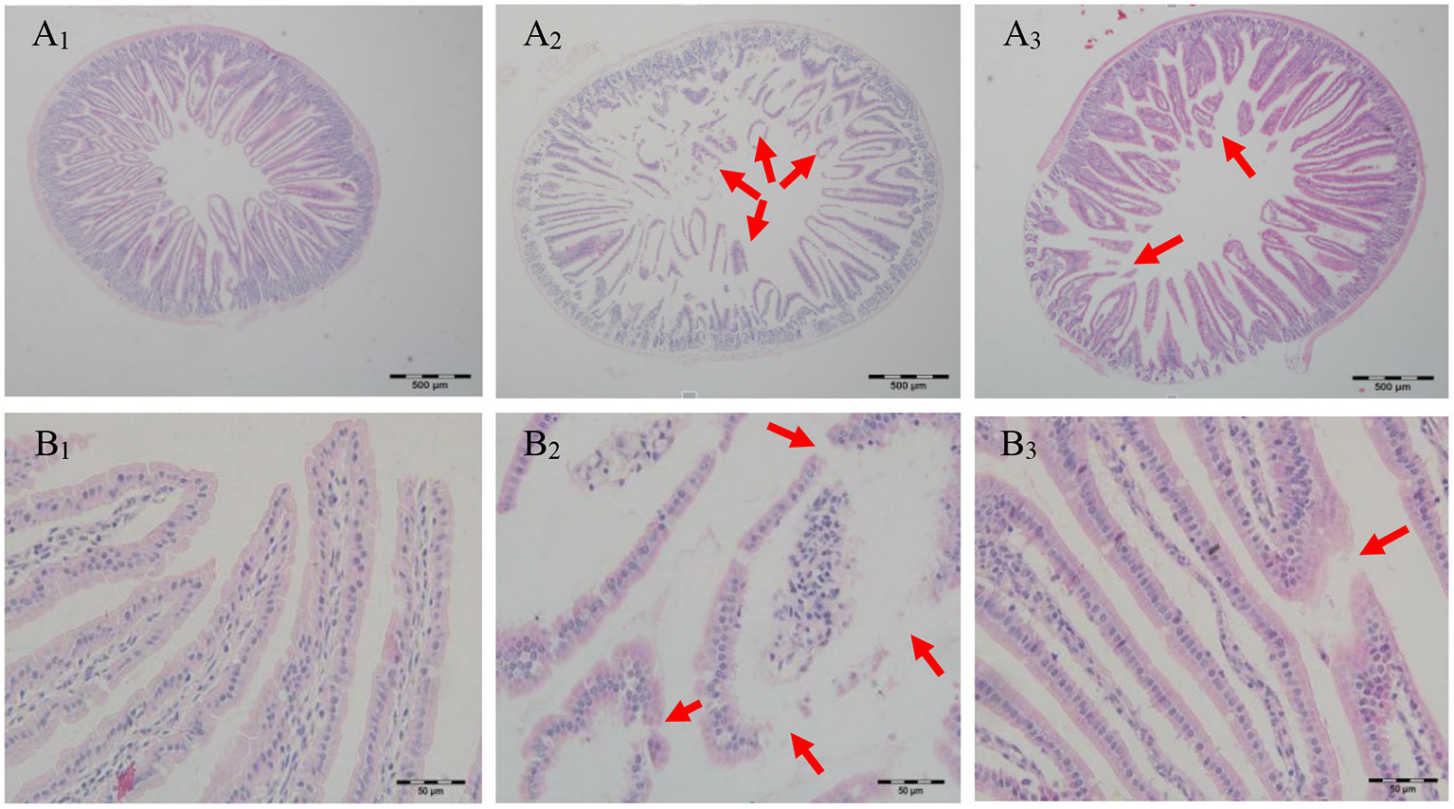
Effect of oral administration with cAMP on jejunal morphology in PN-sensitized mice. Jejunum samples were fixed in 10% buffered formalin and embedded in paraffin. 5 μm sections were stained with H&E and then the sections were examined under light microscopy at 40 **(A)** or 400X **(B)** magnification: (1) the CK group, (2) the PN group, and (3) the cAMP group. The red arrow indicates typical lesions.

Food allergy was associated with defect of the intestinal barrier function and antigen penetrated across the dysfunctional intestinal barrier leading to recruitment of mast cells, production of allergen-specific IgE, and secretion of T-helper 2 (Th2)-related cytokines, eventually causing the food allergy (39). To some extent, intestinal morphology can be an indicator of intestinal structure and function (40). Few studies have reported the effect of cAMP on jejunal morphology in PN-sensitized mice. As seen in Figure 4, villi structure of the jejunum in the CK group was tidy and intact. However, jejunum from PN-sensitized mice had severe morphological structural changes and its villi structure was destroyed and most of villi were atrophied, broken, and shed. In contrast, villi structure of the jejunum in cAMP-treated mice was complete and had just a slight swelling. Morphological analysis showed that the impaired jejunal structure observed in the PN group was largely attenuated by repeated oral administration of cAMP, suggesting that the effect of cAMP on the intestinal barrier function contributes to its amelioration of peanut allergy.

#### Restored Spleen Index

**Figure 5:**
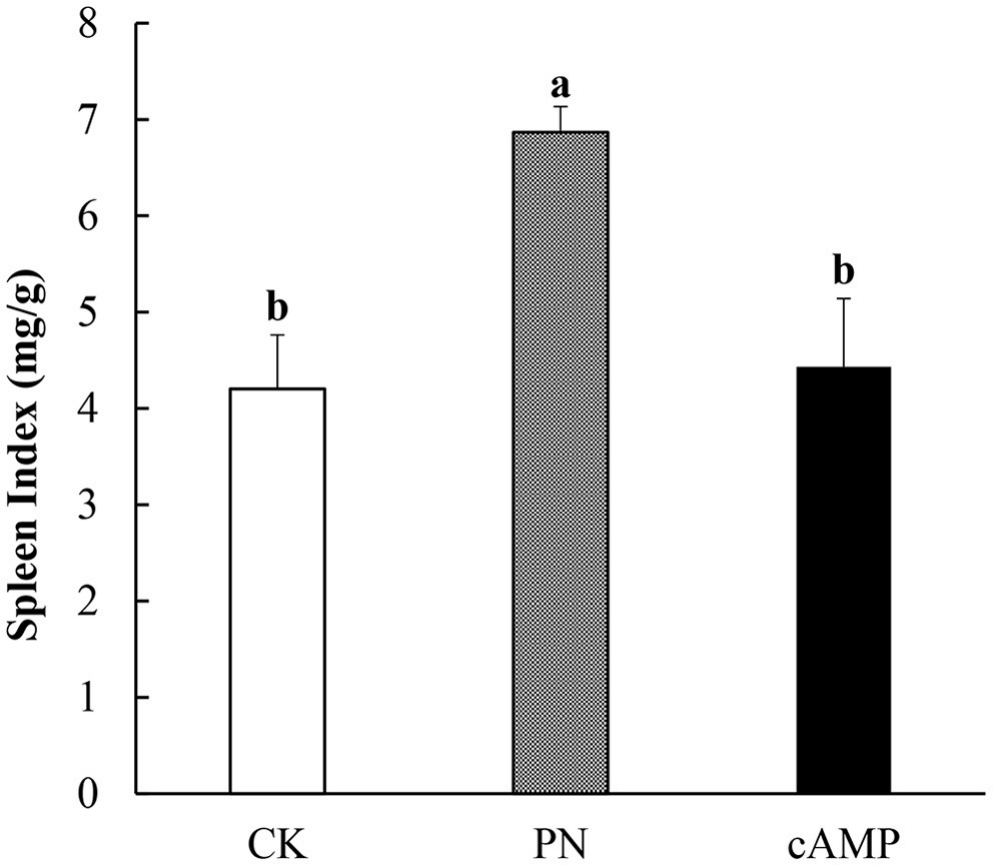
Effect of oral administration with cAMP on spleen index in PN-sensitized mice. Data are expressed as the means ± SDs. Different lowercase letters represent mean significant difference (*p* < 0.05) between the groups of mice.

To systematically investigate the effect of cAMP on PN-sensitized mice, not only is morphological structure of the jejunum examined, so is the spleen. As an important immune organ, the spleen plays an important role in mediating immune responses. When antigen appears in the blood, macrophages and lymphocytes in the spleen will have an immune response and swallow antigen, resulting in splenomegaly. Therefore, the changes in the spleen shape and spleen index can be used as an indicator to identify the sensitization effect (41). As shown in Figure 5, the spleen index in the PN group was significantly increased compared with that in the CK group. It indicated that the mice in the PN group had a severe allergic reaction, leading to significant splenomegaly (Figure 5). However, the spleen index was significantly reduced after PN-sensitized mice were treated with cAMP. The results showed that cAMP accelerated the recovery of spleen index in the PN-sensitized mice, which indicated that cAMP treatment contributed to establish immune homeostasis in physiological systems.

#### Inhibited Specific Immunoglobulin E and Histamine Levels

**Figure 6:**
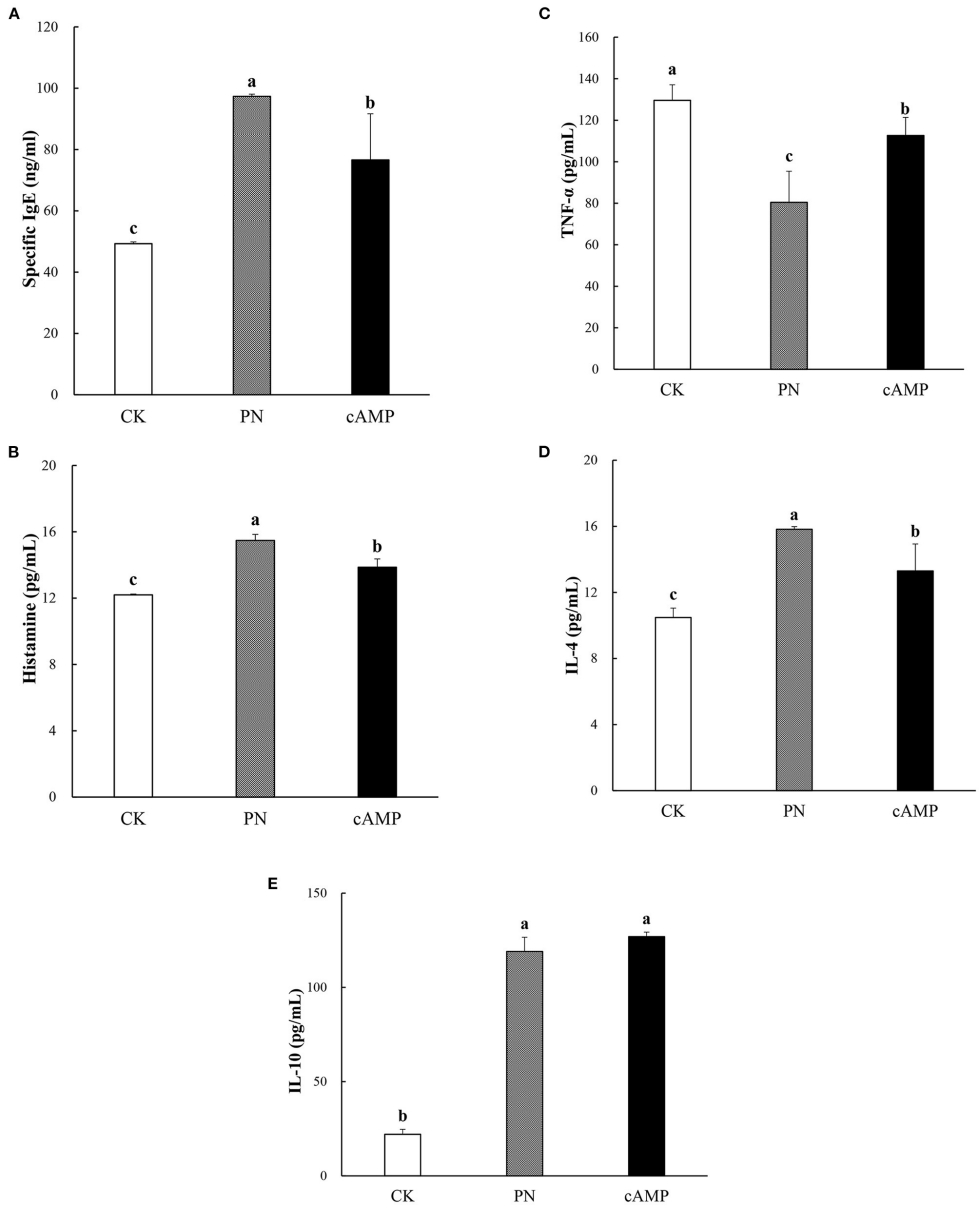
Effect of oral administration with cAMP on serum-specific immunoglobulin (IgE) **(A)**, histamine **(B)**, tumor necrosis factor-α (TNF-α) **(C)**, interleukin-4 (IL-4) **(D)**, and interleukin-10 (IL-10) **(E)** levels in PN-sensitized mice. The levels of these biological markers were measured by ELISA after repeated oral administration of cAMP. Data are expressed as the means ± SDs. Different lowercase letters represent mean significant difference (*p* < 0.05) between the groups of mice.

Peanut allergy is an IgE-mediated type I hypersensitivity (42). The specific IgE produces after exposure to allergens, which triggers mast cell degranulation (43). Activated mast cells degranulate to release different kinds of mediators, such as histamine, and many other cytokines and chemokines, and then symptoms of immediate hypersensitivity occurred (44). In this study, we investigated whether oral administration of cAMP suppressed the increase in specific IgE or histamine levels in serum of PN-sensitized mice. Consistent with the clinical symptoms, serum-specific IgE and histamine levels of mice in the PN group were both significantly higher compared to those in the CK group, whereas the specific IgE and histamine levels in the cAMP group were both significantly reduced compared with the levels in the PN group (Figures 6A,B). Our data showed that repeated oral administration of cAMP significantly reduced the serum-specific IgE and histamine levels in PN-sensitized mice.

#### Effect of Cyclic Adenosine Monophosphate Treatment on T-Helper 1/T-Helper 2 Cytokines in Peanut-Sensitized Mice

Allergy and inflammation are associated with enhanced production of Th2-associated cytokines (Such as, IL-4 and IL-10) and decreased production of Th1-associated cytokines (Such as, IFN-γ and TNF-α) (45). Thus, we investigated the levels of cytokines in a mouse model of peanut allergy. As a result, PN-sensitized mice showed a marked increase in IL-4 and decrease in TNF-α as compared with the CK group (Figures 6C,D). Likewise, mice sensitized by PN showed a significant increase in the secretion of IL-10 (Figure 6E). In contrast, treatment with cAMP potently decreased the levels of IL-4 (Figure 6D) and caused a significant increase of TNF-α (Figure 6C) in the serum compared to the PN group. However, the serum levels of IL-10 in mice were not remarkably affected by the cAMP treatment (Figure 6E).

The Th1/Th2 balance is considered to play an important immunomodulatory role in allergic reactions (46). Naive T cells differentiation to Th2 cells can be promoted by IL-4 and it is believed that an increase in IL-4 shifts the Th1/Th2 balance to predominantly Th2 (47). Thus, cAMP may inhibit the production of IL-4, leading to downregulation of the Th2 response. The Th1/Th2 balance bias toward Th1 is also reflected in the suppression of IgE production (46, 48). Previous studies have shown that cAMP treatment restored the ratio of IFN-γ/IL-4 and normalized the differentiation of Th1/Th2 (28). This study corroborates the results of previous studies, suggesting that cAMP may regulate the Th1/Th2 balance to play an anti-allergic role.

However, cytokines (IL-4, IL-10, TNF-α, and so on) related to allergy action were extremely complex, for example, IL-10 can be produced by the wide variety of innate and adaptive immune cells and IL-10 secretion is susceptible to environmental factors (49). Therefore, it may be hypothesized that environmental stimuli should be responsible for the IL-10 levels since no significant change occurs after cAMP treatment in PN-sensitized mice in this study. Further studies, however, are needed to better elucidate the interaction mechanism.

## Conclusion

In this study, the extraction of cAMP from jujube by UHPE was optimized by OAD and the yield of the extracts was evaluated. The optimal conditions to obtain the highest cAMP yield of jujube by UHPE were determined to be 300 MPa and 20 min, with a 2.5-ml/g liquid-to-solid ratio. Based on the results, we concluded that UHPE is a promising technique for extracting cAMP from jujube, which might play an important role in improving the extraction yield of cAMP and sustainably using jujube resources. Meanwhile, cAMP has an anti-allergic effect in PN-induced allergy. The effect was associated with alleviated clinical symptoms, improved spleen and jejunal morphology, suppression of specific IgE and histamine release, and regulation of IL-4 and TNF-α levels. However, further studies are needed to clarify the anti-allergic effect and mechanism.

## Data Availability Statement

The raw data supporting the conclusions of this article will be made available by the authors, without undue reservation.

## Ethics Statement

The animal study was reviewed and approved by Northwest A&F University Animal Care and Use Committee.

## Author Contributions

CS: methodology, validation, investigation, software, and writing. QB: visualization and investigation. XF: data curation and writing. CW: investigation. YL: funding acquisition. ZG: supervision and funding acquisition. FL: conceptualization, supervision, and funding acquisition.

## Funding

This study was supported by the National Natural Science Fund (No. 31601395), the Open Research Fund Program of Beijing Key Laboratory of Plant Resource Research and Development, Beijing Technology and Business University (PRRD-2021-YB8), the Key Program for Shaanxi Science and Technology (No. 2020NY-146), and the Lueyang Black-Bone Chicken Industry Development Research Institute (WJYJY-2021-9).

## Conflict of Interest

The authors declare that the research was conducted in the absence of any commercial or financial relationships that could be construed as a potential conflict of interest. The reviewer LS declared a shared affiliation with the authors to the handling editor at time of review.

## Publisher's Note

All claims expressed in this article are solely those of the authors and do not necessarily represent those of their affiliated organizations, or those of the publisher, the editors and the reviewers. Any product that may be evaluated in this article, or claim that may be made by its manufacturer, is not guaranteed or endorsed by the publisher.
